# Oral Colonization of *Staphylococcus* Species in a Peritoneal Dialysis Population: A Possible Reservoir for PD-Related Infections?

**DOI:** 10.1155/2018/5789094

**Published:** 2018-08-02

**Authors:** Liliana Simões-Silva, Susana Ferreira, Carla Santos-Araujo, Margarida Tabaio, Manuel Pestana, Isabel Soares-Silva, Benedita Sampaio-Maia

**Affiliations:** ^1^i3S-Instituto de Investigação e Inovação em Saúde, Universidade do Porto, Porto, Portugal; ^2^INEB-Instituto de Engenharia Biomédica, Universidade do Porto, Rua Alfredo Allen 208, 4200-180 Porto, Portugal; ^3^Faculdade de Medicina, Universidade do Porto, Porto, Portugal; ^4^Faculdade de Medicina Dentária, Universidade do Porto, Porto, Portugal; ^5^Departamento de Nefrologia, Centro Hospitalar de São João, EPE, Porto, Portugal; ^6^Departmento de Fisiologia e Cirurgia Cardiotorácica, Faculdade de Medicina, Centro de I&D Cardiovascular, Universidade do Porto, Porto, Portugal; ^7^Departmento de Doenças Renais, Urológicas e Infecciosas, Faculdade de Medicina, Universidade do Porto, Porto, Portugal

## Abstract

Peritoneal dialysis-related infections are important morbidity/mortality causes, being staphylococci the most prevalent agents. Since *Staphylococcus aureus* nasopharynx carriage is a known risk factor for PD infections and the oral cavity is a starting point for systemic diseases development, we aimed at comparing the oral staphylococci colonization between PD patients and controls and studying the association with PD-related infections. Saliva samples were plated in Mannitol salt, and isolates were identified by *DnaJ* gene sequencing. Staphylococci PD-related infections were recorded throughout the 4-year period following sample collection. *Staphylococcus* colonization was present in >90% of the samples from both groups (a total of nine species identified). PD patients presented less diversity and less prevalence of multispecies *Staphylococcus* colonization. Although all patients presenting *Staphylococcus epidermidis* PD-related infections were also colonized in the oral cavity by the same agent, only 1 out of 7 patients with ESI caused by *S*. *aureus* presented *S. aureus* oral colonization. Staphylococci are highly prevalent in the oral cavity of both groups, although PD patients presented less species diversity. The association between oral *Staphylococcus* carriage and PD-related infections was present for *S. epidermidis* but was almost inexistent for *S. aureus*, so, further studies are still necessary to evaluate the infectious potential of oral *Staphylococcus* carriage in PD.

## 1. Introduction

Peritoneal dialysis (PD) is a home-based renal replacement therapy for end-stage chronic kidney disease (CKD) patients, presenting significantly lower costs and higher satisfaction levels in comparison to hemodialysis (HD) [1]. Nonetheless, infection remains an important cause of morbidity and mortality among PD patients [1, 2]. Peritonitis and exit-site infections (ESI) are the more relevant and common PD-related infections, and *Staphylococcus* species are the most frequent etiological agents responsible for both types of infection [3, 4]. Worldwide, *Staphylococcus epidermidis* and other coagulase-negative *Staphylococcus* (CNS) are the most prevalent PD-related infectious agents. On the contrary, *Staphylococcus aureus* is associated with more severe PD-related infections leading to increased risks of hospitalization, death, and catheter removal [2, 5, 6]. *Staphylococcus haemolyticus, Staphylococcus warneri, Staphylococcus hominis, Staphylococcus simulans, Staphylococcus capitis*, and *Staphylococcus saprophyticus* are staphylococci species also reported as PD-related infectious agents [7, 8].

It is assumed that contamination at the time of PD fluid exchange is a major cause of peritonitis, occurring mainly by an external route [9]. For this reason, general measures concerning exit-site care, such as meticulous hand hygiene and face-mask wear are recommended during the dialysis exchange by the International Society for Peritoneal Dialysis (ISPD) [10]. Other measure is the prophylactic use of topical antibiotic treatment of PD catheter exit-site, known to reduce ESI [4, 11], although its effect on peritonitis rate is not clear [4, 11, 12]. Also, the screening for nasal *S. aureus* carriage prior to PD catheter insertion is recommended and, if positive, treatment with topical nasal application of mupirocin is recommended [13]. The efficacy of this prophylactic intranasal antibiotic treatment has been showed in several prospective studies [14–16].

Another less explored route of infection in PD patients is the haematogenous pathway. It is known that oral microorganisms are frequently responsible for bacteraemia due to routine daily hygiene activities, such as tooth brushing and dental tape use or invasive dental procedures [17, 18]. For that reason, risk patients including those on PD therapy should receive prophylactic antibiotic treatment before dental interventions [17]. Although it is recognized that the oral cavity can be a starting point for dissemination of pathogenic organisms, by both external and haematogenous routes, to our knowledge, there are no studies linking the oral microbiome and PD-related infections. Thus, the aim of the present study was to assess the oral colonization with *Staphylococcus* spp. in PD patients in comparison with healthy controls, and explore in patients on PD therapy the association between oral colonization with *Staphylococcus* spp. and PD-related infections.

## 2. Materials and Methods

Twenty-one end-stage CKD adult patients undergoing PD therapy followed up at the outpatient clinic of the Nephrology Department of the “Centro Hospitalar de S. João” for more than one month were invited to participate in the study. A convenience sample was obtained related with the attendance of patients to the outpatient clinic during a period of 6 months. The control group included 14 adult healthy subjects recruited among family members of PD patients, to gather individuals with the same socioeconomic and environmental conditions as PD patients. The exclusion criteria were recent history of infection (less than 1 month), inability to give informed consent, pregnancy, and severe acute illness. The study protocol was approved by the Ethics Committee for Health and Institutional Review Board of São João Hospital Centre.

Demographic and relevant clinical information was gathered for each patient including age, gender, smoking habits, education level, blood pressure, aetiology of renal disease, residual renal function, time on PD, and infectious complications during PD. Demographic information was also gathered for the control healthy population, namely, age, gender, smoking habits, and education level.

### 2.1. Oral Evaluation

Oral hygiene was assessed in both groups using the visual plaque indexes (VPI) in four sites of each tooth (mesiobuccal, midbuccal, distobuccal, and midlingual); the percentage of the examined sites with visible plaque ranged from 0% to 100%.

Additionally, before the oral clinical evaluation, unstimulated whole saliva was collected from both groups into a sterile container for 5 minutes in a quiet room, between 9:00 to 11:00 AM to minimize the circadian rhythm effects and at least 2 h after eating, tooth brushing, mouth washing, or smoking. Immediately after collection, the saliva was stored at −20°C for biochemical analysis or mixed 1 : 1 in Brain Heart Infusion with 20% glycerol for microbial analysis and cryopreserved at −80°C. The total volume collected over a 5 min period was registered, for saliva flow rate (mL/min) determination. The saliva pH was also measured immediately after collection using pH strips (5.0–8.0, Duotest, Germany). The saliva urea concentration was determined by an enzymatic UV test (method “urease-GLDH”) and quantified by an automatic analyser, Pentra C200 (Horiba ABX Diagnostics, Switzerland).

### 2.2. *Staphylococcus* spp. Isolation

*Staphylococcus* was isolated from saliva from both groups and quantified. Saliva samples were serially diluted with 0.9% sterile NaCl solution and plated in triplicate in a selective and differential culture medium, Mannitol salt agar (Liofilchem, Italy). Plates were incubated aerobically for 48 h at 37°C. The total number of colonies was determined, and the quantification results were expressed in a logarithmic scale of colony-forming units per ml of saliva (log_10_CFU/ml). The lower limit of detection was 10^2^ CFU/ml. The Gram-positive and catalase-positive isolates were purified by reisolation in BHI (Biolab Inc, Budapest, Hungary) agar and stored in BHI containing 10% glycerol at −80°C. The isolated colonies were identified using the *DnaJ* gene amplification and sequencing approach.

### 2.3. *Staphylococcus* Identification by *DnaJ* Gene Amplification

DNA was extracted using the boiling method; briefly, 5 colonies were suspended in 100 *μ*L of molecular grade water and incubated at 100°C for 10 minutes. The diluted suspension (1 : 10) was centrifuged at 12,000 rpm for 10 min, and the supernatant was used as a DNA template. *DnaJ* gene was amplified with the DFS Master Mix (Bioron, Germany) according to the manufacturer's instructions using the primers Staph_forward, 5′-GCCAAAAGAGACTATTATGA-3′, and Staph_reverse, 5′-ATTGYTTACCYGTTTGTGTACC-3′ and the amplification conditions previously described, with MyCycler Thermal Cycler (Bio-Rad, California, USA) [19]. PCR products were visualized by electrophoresis on a 1% agarose gel (SeaKem® LE Agarose; Lonza, Cologne, Germany) with GelRed^TM^ (GeneON, Ludwigshafen am Rhein, Germany) and then visualized in ChemiDoc XR (Bio-Rad, California, USA). PCR amplification products were identified by sequencing (StabVida, Caparica, Portugal). The identification by sequencing of *DnaJ* gene was only considered when the sequencing identity was 98% or higher to *DnaJ* sequences or sequences resulting from whole genome sequencing deposited at GenBank [20].

### 2.4. PD-Related Infection Episodes

The PD-related infections by *Staphylococcus* spp. occurring from the start of PD therapy up to 4 years after sample collection were registered in all 21 studied PD patients. However, due to PD technique dropout, 20 patients remained in the first follow-up year, 19 in the second, 14 in the third, and 11 in the fourth.

### 2.5. Data Analysis

Data were analysed using IBM SPSS Statistics for Windows, Version 23.0 (IBM Corp, NY, USA). The categorical variables were described through relative frequencies (%) and analysed by the chi-square independence test or Fisher Exact test when more than 1 cell had expected counts less than 5. Continuous variables were described using mean ± standard deviation (SD) and analysed by Student's *t*-test. A *P* value of less than 0.05 was assumed to denote a statistically significant difference.

## 3. Results

Clinical information of PD patients is included in Table 1, namely, aetiology of CKD, PD vintage, residual renal function, and blood pressure. The most prevalent aetiologies of CKD were glomerular disease, including diabetic nephropathy, and tubulointerstitial disease, namely, polycystic kidney disease. At sample collection, the average time on PD therapy was 15.45 ± 16.90 months, ranging from 1 to 72 months.

Demographic information, namely, age, gender, educational level, smoking habits, parameters regarding oral hygiene, and saliva biochemistry of both groups is included in Table 2. The two groups presented similar demographic characteristics. Also, there were no significant differences between the two groups in the education level and smoking habits. Both groups had high VPI values, revealing a low oral hygiene status. Regarding saliva biochemical parameters, the only significant differences were in the saliva pH and urea concentration, being both parameters higher in PD patients when compared to the control group.

The PD patients and healthy controls presented similar total load (CFU/mL) of *Staphylococcus* in saliva (Table 3). Nine *Staphylococcus* species were identified in the saliva from both groups, namely, *S. epidermidis, S. aureus, S. capitis, S. saprophyticus*, *S. hominis, Staphylococcus cohnii*, *Staphylococcus pasteuri*, *Staphylococcus lugdunensis*, and *S. warneri* (Supplementary Table 1).

No significant differences were observed in the prevalence of the different *Staphylococcus* species between the two groups. However, the prevalence of more than one *Staphylococcus* species (multispecies) in saliva was significantly higher in the control group than in PD patients (Table 4). When analyzing the colonization status of the family members of PD patients (10 pairs), we found that 6 patient-family member pairs presented oral colonization by *S. epidermidis*. One of these pairs also presented colonization by *S. saprophyticus*.

The occurrence of PD-related *Staphylococcus* infectious episodes was retrieved from clinical records from the date of entrance in the PD program until PD dropout or the end of the 4th year after oral sample collection, accounting for an average of 53.9 ± 27.8 months. The total number of patients that had at least one infection episode, peritonitis and/or ESI, is presented in Table 5, whereas in Table 6, the total number of infection episodes and the *Staphylococcus* species detected are presented.

**Figure 1:**
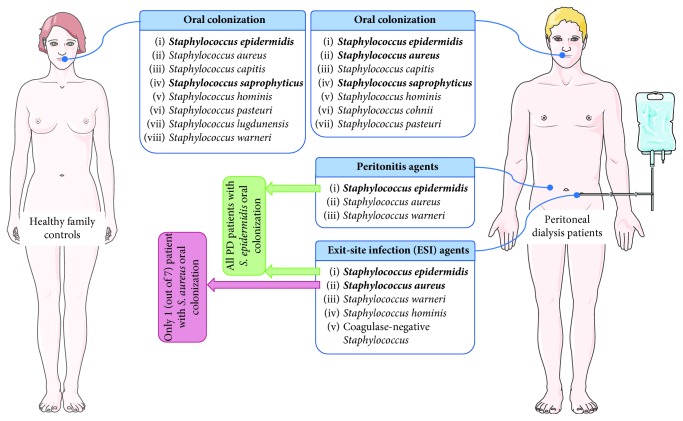
*Staphylococcus* oral colonization of PD patients and healthy controls and *Staphylococcus* agents responsible for PD-related infections. Common species are in bold. Figure was produced using Servier Medical Art, http://www.servier.com/Powerpoint-image-bank.

The association between the PD-related infections by staphylococci and the oral colonization with the same *Staphylococcus* species was examined (Figure 1). All PD patients with peritonitis or ESI caused by *S. epidermidis* were colonized by the same species in the oral cavity (Table 7). Among the PD patients with ESI caused by *S. aureus*, only 1 was colonized with the same species in the oral cavity (Table 7). Moreover, the only patient with *S. aureus* peritonitis was also not orally colonized with the same species. Oral colonization of PD patients with multispecies staphylococci was not associated to a higher prevalence of PD-related infections (*P* > 0.05).

## 4. Discussion

In the Human Oral Microbiome Database (http://www.homd.org/), few *Staphylococcus* species are described, namely, *S. aureus*, *S. epidermidis*, *S. warneri*, and *Staphylococcus caprae.* Our results provide evidence for the presence of six other *Staphylococcus* species, namely, *S. saprophyticus*, *S. capitis*, *S. cohnii*, *S. pasteuri*, *S. hominis*, and *S. lugdunensis*. Taking in consideration that we found a prevalence close to 100% of *Staphylococcus* in both PD patients and control groups, we suggest that this genus is a common member of the oral microbiome. In agreement, other authors also described *Staphylococcus* species as frequent oral colonizers of adults, in health and oral disease [21, 22]. Nonetheless, our study was the first to report *S. pasteuri*, considered a rare CNS clinical isolate [23], as a member of the oral microbiome.

Although staphylococci are major pathogens of PD-related infections, there are no studies exploring the oral microbiome as a source of infection in PD patients. The likelihood that these microorganisms could act as opportunistic infectious agents to distant body locations triggered our need to better understand the oral colonization status of PD patients. Overall, we did not observe differences between the two groups in specific species oral colonization, although the oral microbiota of PD patients presented lower staphylococci diversity. This lower diversity in PD patients may be justified by more frequent antibiotic therapy of these patients, as well as by changes in the oral biochemical milieu, including higher pH and urea content observed in patients on PD therapy.

Although infections are a major concern to PD patients, risk factors and transmission routes are far from being fully understood. Given that many preventive measures are in place to prevent PD-related infections by external route, such as mask use and prophylactic use of topical antibiotic treatment of PD catheter exit-site [4, 10, 11], staphylococci transmission by a haematogenous route should be considered. In line with this, recent studies demonstrate that the uterine microbiome is more similar to the oral microbiome than to the vaginal, intestinal, or skin microbiome [24, 25], which reinforces the role of the haematogenous route in the transmission of oral microorganisms.

In agreement with previous reports, the *Staphylococcus* species identified as agents of PD-related infections in our group of PD patients were *S. aureus*, *S. epidermidis*, *S. warneri*, and *S. hominis* [7, 8, 26]. In the present study, all the patients with *S. epidermidis* infections, either peritonitis or ESI, were colonized orally by the same species suggesting that the oral cavity may have the potential to behave as a reservoir of PD-related infections induced by these agents. However, *S. epidermidis* is a highly ubiquitous microorganism in human skin and mucosa, and so, the phylogenetic relatedness between common species would be an important asset to determine the real transmission route. Moreover, only 1 out of 7 patients with *S. aureus* ESI was colonized orally by *S. aureus* thus suggesting that the oral cavity may not behave as a potential reservoir of PD-related infections induced by *S. aureus* specifically. These partial contradictory results, together with the small sample size restricted to a single-centre, hinder us to draw a conclusion about the potential of the oral cavity as a reservoir of PD-related pathogens. Further studies are thus necessary to clarify this hypothesis.

## 5. Conclusion

Staphylococci are highly prevalent in the oral cavity of both groups, although PD patients presented less diversity of species in comparison to controls. Given that the association between oral *Staphylococcus* carriage and PD-related infections was present for *S. epidermidis* but was almost inexistent for *S. aureus*, further studies are necessary to evaluate the relationship between oral *Staphylococcus* carriage and PD-related infections development.

## Figures and Tables

**Table 1:** Aetiology of chronic kidney disease (CKD), peritoneal dialysis (PD) vintage, residual renal function, and blood pressure of PD patients.

	PD patients
Aetiology of CKD	
Glomerular disease	52.38%
Diabetic nephropathy	19.05%
Other glomerular diseases	33.33%
Tubulointerstitial disease	23.81%
Autosomal dominant polycystic kidney disease	14.29%
Other tubulointerstitial diseases	9.52%
Unknown	23.81%
PD vintage (months)	15.45 ± 16.90
Residual renal function (mL/min)	6.98 ± 4.52
Blood pressure	
Systolic	130.19 ± 19.65
Diastolic	78.29 ± 10.96

Results are shown in prevalence (%); CKD, chronic kidney disease; PD, peritoneal dialysis.

**Table 2:** Demographic information and parameters regarding oral hygiene of peritoneal dialysis (PD) patients and healthy controls.

	PD patients	Controls	*P* value
Age (years)	46.8 ± 9.7	42.2 ± 14.5	0.282
Sex (male %)	42.9%	28.6%	0.392
Education level			>0.999
Basic/elementary school	85.7%	83.3%	
High school/university	14.3%	16.7%	
Smoking habits			
Past	58.3%	33.3%	0.387
At sample collection	16.7%	11.1%	>0.999
Visual plaque index (%)	59.7 ± 27.7	48.4 ± 23.1	0.254
Saliva biochemistry			
Flow rate (mL/min)	0.40 ± 0.26	0.41 ± 0.30	0.871
pH	7.72 ± 0.53	7.06 ± 0.39	**<0.001**
Urea (mg/dL)	110.41 ± 36.64	28.79 ± 5.90	**<0.001**

Results are shown in prevalence (%) or mean ± SD; PD, peritoneal dialysis.

**Table 3:** Prevalence and quantification of *Staphylococcus* spp. in the oral cavity of PD patients and controls.

	PD patients	Controls	*P* value
Prevalence	90.5%	92.9%	>0.999
Counts (log_10_CFU/mL)	2.79 ± 0.60	2.43 ± 0.64	0.120

Results are prevalence (%) or mean ± SD; PD, peritoneal dialysis; CFU, colony-forming units.

**Table 4:** Prevalence of *Staphylococcus* species identified in isolates from saliva of peritoneal dialysis (PD) patients and controls.

	PD patients	Controls	*P* value
*Staphylococcus species*			
*S. epidermidis*	89.5%	92.3%	>0.999
*S. aureus* spp. *aureus*	21.1%	23.1%	>0.999
*S. capitis*	15.8%	7.7%	0.629
*S. saprophyticus*	10.5%	15.4%	>0.999
*S. hominis*	5.3%	23.1%	0.279
*S. cohnii* spp. *cohnii*	5.3%	0%	>0.999
*S. pasteuri*	5.3%	7.7%	>0.999
*S. lugdunensis*	0%	7.7%	0.406
*S. warneri*	0%	15.4%	0.157
Multispecies	36.8%	76.9%	**0.026** ^*∗*^

Results are prevalence (%); PD, peritoneal dialysis; ^*∗*^ *P* < 0.05.

**Table 5:** Total number of PD patients with one or more infectious episodes, peritonitis, and/or exit-site infections (ESI), in particular caused by *Staphylococcus* spp.

	Peritonitis		ESI	
	Total	*Staphylococcus* spp.	Total	*Staphylococcus* spp.
PD patients with infectious episodes (*n*=19)	10 (47.6%)	5 (23.8%)	19 (90.5%)	12 (57.1%)

**Table 6:** Total number of infectious episodes and *Staphylococcus* species identified in peritonitis and exit-site infections (ESI) of PD patients.

Infectious agent	PD-related infections (*n*=114)	
	Peritonitis (*n*=23)	ESI (*n*=91)
Others	16 (69.6%)	63 (69.2%)
Staphylococci	**7 (30.4%)**	**28 (30.8%)**
*S. epidermidis*	5	9
*S. aureus*	1	16
*S. warneri*	1	1
*S. hominis*	0	1
Nonidentified CNS	0	1

CNS, coagulase-negative *Staphylococcus*.

**Table 7:** Comparison between peritoneal infections, peritonitis, or exit-site infections (ESI) caused by *Staphylococcus* species and oral colonization of PD patients.

	Infectious agent		Oral colonization	
	ESI	Peritonitis	ESI	Peritonitis
*Staphylococcus epidermidis*	6	3	6	3
*Staphylococcus aureus*	7	1	1	0

## Data Availability

The data used to support the findings of this study are available from the corresponding author upon request.

## Supplementary Materials

Supplementary Materials Supplementary Table 1 presents the *Staphylococcus* species identified in PD patients and controls, as well as the number of individuals where each of the *Staphylococcus* species was identified.
