# Supplementary material for: Oral Colonization of Staphylococcus Species in a Peritoneal Dialysis Population: A Possible Reservoir for PD-Related Infections?
Source: Can J Infect Dis Med Microbiol. 2018 Aug 2;2018:5789094. doi: 10.1155/2018/5789094 (PMC6098888; doi:10.1155/2018/5789094)
Supplement: Supplementary Materials — Supplementary Table 1 presents the Staphylococcus species identified in PD patients and controls, as well as the number of individuals where each of the Staphylococcus species was identified. [file 5789094.f1.pdf]

Supplementary Table 1: *Staphylococcus* species identified in peritoneal dialysis (PD) patients and controls. n reports to the number of individuals, PD patients and controls, where each of the *Staphylococcus* specie was identified.

<table>
<tr><th></th><th></th><th>Staphylococcus species (n)</th><th>Acession number</th></tr>
<tr><td rowspan="3"></td><td rowspan="8">PD patients</td><td>S. saprophyticus subsp. bovis (1)</td><td>AB234088.1</td></tr>
<tr><td>S. capitis subsp. urealyticus (3)</td><td>AB234061.1</td></tr>
<tr><td>S. cohnii subsp. cohnii (1)</td><td>AB234066.1</td></tr>
<tr><td rowspan="9">Controls</td><td>S. epidermidis (30)</td><td>AB234069.1</td></tr>
<tr><td>S. aureus subsp. aureus (8)</td><td>AB234058.1</td></tr>
<tr><td>S. pasteuri (2)</td><td>CP004014.1</td></tr>
<tr><td>S. saprophyticus subsp. saprophyticus (4)</td><td>AB234089.1</td></tr>
<tr><td>S. hominis subsp. novobiosepticus (2)</td><td>AB234076.1</td></tr>
<tr><td rowspan="4"></td><td>S. capitis subsp. capitis (1)</td><td>AB234060.1</td></tr>
<tr><td>S. hominis subsp. hominis (2)</td><td>AB234075.1</td></tr>
<tr><td>S. lugdunensis (1)</td><td>AB234081.1</td></tr>
<tr><td>S. warneri (2)</td><td>AB234328.1</td></tr>
</table>
